# Superiority Verification of Deep Learning in the Identification of Medicinal Plants: Taking *Paris polyphylla* var. *yunnanensis* as an Example

**DOI:** 10.3389/fpls.2021.752863

**Published:** 2021-09-22

**Authors:** JiaQi Yue, WanYi Li, YuanZhong Wang

**Affiliations:** ^1^Medicinal Plants Research Institute, Yunnan Academy of Agricultural Sciences, Kunming, China; ^2^College of Traditional Chinese Medicine, Yunnan University of Chinese Medicine, Kunming, China

**Keywords:** deep learning, identification research, medicinal plant, *Paris polyphylla* var. *yunnanensis*, superiority verification, ResNet

## Abstract

Medicinal plants have a variety of values and are an important source of new drugs and their lead compounds. They have played an important role in the treatment of cancer, AIDS, COVID-19 and other major and unconquered diseases. However, there are problems such as uneven quality and adulteration. Therefore, it is of great significance to find comprehensive, efficient and modern technology for its identification and evaluation to ensure quality and efficacy. In this study, deep learning, which is superior to conventional identification techniques, was extended to the identification of the part and region of the medicinal plant *Paris polyphylla* var. *yunnanensis* from the perspective of spectroscopy. Two pattern recognition models, partial least squares discriminant analysis (PLS-DA) and support vector machine (SVM), were established, and the overall discrimination performance of the three types of models was compared. In addition, we also compared the effects of different sample sizes on the discriminant performance of the models for the first time to explore whether the three models had sample size dependence. The results showed that the deep learning model had absolute superiority in the identification of medicinal plant. It was almost unaffected by factors such as data type and sample size. The overall identification ability was significantly better than the PLS-DA and SVM models. This study verified the superiority of the deep learning from examples, and provided a practical reference for related research on other medicinal plants.

## Introduction

Medicinal plants are a kind of highly exploitable plants with various values such as medicinal edible ecology. Their research has become the latest source for the emergence of new drugs (Newman and Cragg, 2015). The development potential of the international market for the utilization of medicinal plants is huge, and countries all over the world generally attach importance to its research in order to better transform and utilize medicinal plants, solve the problem of human survival resource shortage, and improve human health (Jamshidi-Kia et al., 2018). Medicinal plants have a wide range of sources. Due to differences in regional natural conditions, climatic conditions, flora and natural resources, they present a unique distribution with great differences in quantity and type (Deng et al., 2016). Many factors have different degrees of influence on the quality of medicinal plants. Therefore, the use of comprehensive, efficient, and modern technical means to clarify the region and part of medicinal plants has far-reaching significance for quality and efficacy.

Traditional identification and evaluation techniques for medicinal plants mainly include the technology of DNA barcoding, macroscopic identification, microscopic identification, chromatography, spectroscopy, etc. (Pang et al., 2011; Pei et al., 2020; Liu et al., 2021). Among them, spectroscopy has the advantages of simplicity, speed, economy, and high throughput, which can fully characterize the chemical information of samples with complex mixed systems (Pasquini, 2018). The identification research of medicinal plants mostly uses spectroscopy combined with chemometrics. Among them, the partial least square discriminant analysis (PLS-DA) and support vector machine (SVM) have excellent performance, and have been successfully applied to the identification and evaluation of a variety of medicinal plants, including species identification, origin identification, age identification, part identification, adulteration identification, etc. (Liu et al., 2020; Shen et al., 2020; Wang et al., 2020) Yang and Wang (2018) compared the effects of PLS-DA and SVM on the identification of *P. polyphylla* var. *yunnanensis* from different regions based on infrared spectroscopy and ultraviolet spectroscopy data. It is found that both models have higher recognition performance, and the accuracy of SVM is higher than that of PLS-DA.

In addition, two-dimensional correlation spectroscopy (2DCOS) is also a powerful tool for identification evaluation. This technology fully combines the advantages of computational chemistry, statistics, spectroscopy and computer science to increase the spectral resolution and enrich the information carried by the spectrum by increasing the dimension (Noda, 1989, 1993). In recent years, reports on the research and application of 2DCOS technology are increasing year by year, covering drug metabolism, drug toxicology, drug structure-activity relationship, traditional Chinese medicine, etc. (Noda, 2004, 2014, 2016; Li et al., 2014). Based on years of research, Sun et al. (2003) wrote a book called *“Atlas of Two-dimensional Correlation Infrared Spectroscopy for Traditional Chinese Medicine Identification,”* which contains the 2DCOS spectra of more than 300 kinds of traditional Chinese medicine, providing a reference for the identification research of related traditional Chinese medicine. However, the artificial identification and analysis of 2DCOS spectra has limitations in time, technology, and experience. Moreover, interdisciplinary research has become a current hot spot and also the trend of future scientific research field. Therefore, it is necessary to combine 2DCOS with more modern, convenient and intelligent technical means of other disciplines to realize the rapid identification of medicinal plants.

Deep learning is the main research method used in the development of artificial intelligence research at the present stage, which has unique advantages in image classification and object recognition (LeCun et al., 2015; Houssein et al., 2021). Combining it with 2DCOS images for the identification of medicinal plants can take advantage of the respective advantages of the two technologies and greatly improve the efficiency of identification and analysis. Deep learning combined with 2DCOS seems to show superior performance in many aspects than traditional spectroscopy combined with chemometrics in identifying medicinal plants (Dong et al., 2020). For example, deep learning can achieve good identification without complex spectral preprocessing, and there is no need to manually extract features in the modeling process, which greatly improves efficiency and reduces various risks caused by human factors (Grinblat et al., 2016). However, these conclusions are all based on theories or the application of a single method, and there has been no actual comparison and discussion on them.

*Paris polyphylla* var. *yunnanensis* (PPY), as the original plant of the precious Chinese medicine Paridis Rhizoma, is a medicinal plant resource with a representative and global influence (Cunningham et al., 2018). In the market, there are more than 80 commonly used Chinese patent medicines with Paridis Rhizoma as the main raw material, and 107 pharmaceutical companies are involved in the production, which are distributed in 23 provinces of China. They have significant clinical efficacy and economic value (Tao et al., 2020). At present, domestic and foreign scholars have conducted a lot of research on PPY, but the research on the resources evaluation is still in a situation where there are results but no conclusions, and they are all based on the traditional medicinal rhizoma. Moreover, studying the above-ground parts of PPY can promote the development and utilization of non-medicinal parts, and improve economic benefits (Zhao et al., 2021). Besides, there is currently no research on the use of deep learning combined with 2DCOS to identify the parts and regions of PPY.

In conclusion, taking PPY as an example, two pattern recognition models of PLS-DA and SVM, and a deep learning model of Residual neural network (ResNet) were established in this study to explore and verify whether deep learning combined with 2DCOS has advantages in the identification of medicinal plant resources. In order to increase comparability and credibility, we simultaneously identified and evaluated PPY samples of different regions and parts. In addition, we also compared the impact of different sample sizes on model identification performance to explore whether the three models are dependent on sample size. This research not only provided a reasonable, standardized, fast and effective method for the identification of regions and parts of PPY, but also verified the superiority of the deep learning model in the identification of medicinal plants and the response of the three models to sample size. This is conducive to the development and utilization of advanced deep learning models such as ResNet in other fields.

## Materials and Methods

### Sample Information

**FIGURE 1:**
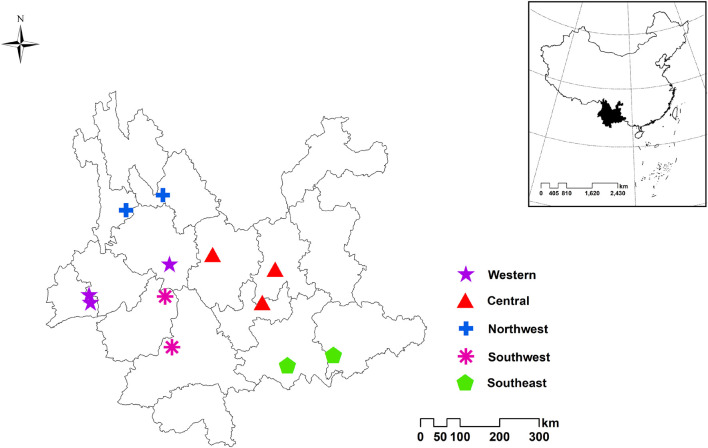
Location distribution of *Paris polyphylla* var. *yunnanensis* samples in western, central, northwest, southwest and southeast of Yunnan.

**FIGURE 2:**
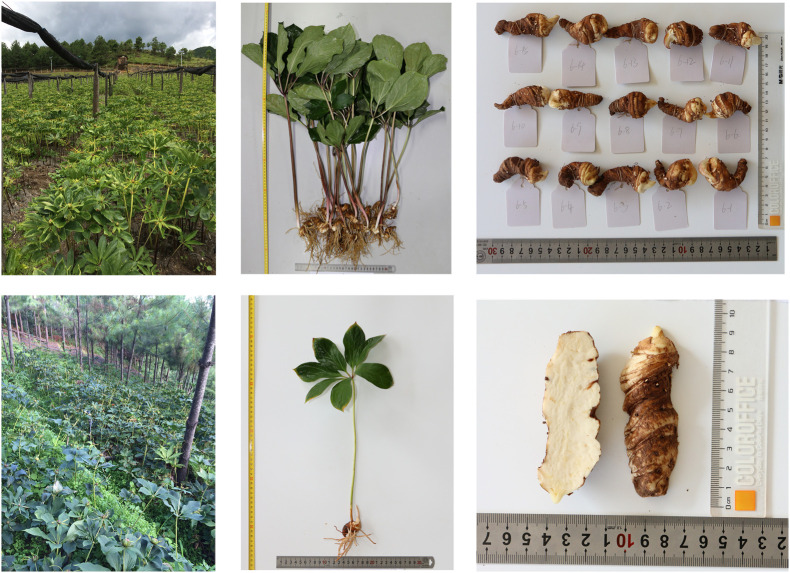
Sample picture of the planting site, whole plant and rhizome of *Paris polyphylla* var. *yunnanensis.*

A total of 772 individuals were collected in 12 sampling sites in central, northwest, southeast, southwest and western Yunnan (Figure 1). All samples were identified as *Paris polyphylla* var. *yunnanensis* by Professor Hang Jin from the Institute of Medicinal Plants, Yunnan Academy of Agricultural Sciences. Some samples are shown in Figure 2. Afterward, all the samples were cleaned and divided into four parts: rhizome, stem, leaf and fibrous root. Then the samples were dried to a constant weight at 50°C in an electric thermostatic drying oven. Next, the samples were passed through a 100-mesh sieve. Finally, the fine powders were stored in self-sealed bags and kept in a dry environment away from light for subsequent analysis. The detailed information of the samples is shown in Supplementary Table 1. There are a total of 772 rhizomes, all of which were used for regions identification analysis. Rhizome (G: 142), stem (J: 107), leaf (Y: 137), and fibrous root (XG: 107) from Dehong and Yuxi were selected for identification of parts.

### FT-MIR Spectra Acquisition

The Fourier transform mid-infrared spectra were collected by a Fourier transform infrared spectrometer equipped with an attenuated total reflection accessory (Perkin Elmer, Norwalk, CT, United States). Sample powder (2 ± 0.2 mg) was placed in the center of the metal ring (ZnSe crystal surface), and the manometer knob was adjusted to a uniform progress bar of 131 ± 1 to form sample powder sheets with the same thickness. The infrared spectrum scanning range was set to be 4,000–550 cm^–1^ with a spectral resolution of 4 cm^–1^. Sixteen times of scanning were carried out, and each sample was measured in parallel for three times. Finally, the average spectrum was taken. Before the sample scanning, the infrared spectrum of the blank crystal surface is collected, and the interference of air and the scattering spectrum of the crystal part was deducted. During the spectrum measurement, keep the laboratory temperature at 25°C and the relative air humidity at 30%.

### Data Processing and Exploratory Analysis

Although the spectral data preprocessing and the characteristic variable selection have been proved by previous studies to be effective for optimizing identification model (Obaid et al., 2019), the complex data preprocessing process will greatly reduce the recognition efficiency. Moreover, the preprocessing methods and characteristic variable selection methods used for different data sets cannot be unified, which requires a lot of time and resource costs to verify. Therefore, this study directly used original spectral data for subsequent identification analysis without considering data preprocessing and characteristic variable selection, so as to fairly compare the recognition performance of the three types of models and verify whether the ResNet model has advantages in the identification research.

In addition, in order to explore the impact of sample size on the recognition ability of the three types of models, we divided the data sets of region and part into low sample size group (10%), medium sample size group (50%), and high sample size group (100%), and the percentage in parentheses is the proportion of each group of samples (Supplementary Table 2). The Kennard-stone algorithm was performed to divide the data of all groups into training set (2/3) and test set (1/3), which was directly used to build PLS-DA and SVM models. The data for establishing the ResNet model is the 2DCOS images of all groups, and the generation method is shown in the following section.

Exploratory analysis used the unsupervised analysis method of t-distributed stochastic neighbor embedding (t-SNE) to summarize the distribution of grouped samples in a multivariate space. By identifying the distribution trend of samples, high-dimensional data can be visualized as data points in two-dimensional or three-dimensional graphs. The above process was completed by MATLAB software.

### Two-Dimensional Correlation Spectroscopy Spectra Image Acquisition

The generalized two-dimensional correlation spectrum is an effective method to improve spectral resolution and solve spectral overlap by designing disturbance variables, which is obtained by discrete generalized 2DCOS algorithm. Its dynamic spectrum is expressed as ***S***, and the expression is as follows, where *v* is variable and *t* is the external disturbance (Noda, 2018).


(1) $$S\left(v\right)=\left[\begin{array}{c} y\left(v,t_{1}\right)\\ y\left(v,t_{2}\right)\\ y\left(v,t_{3}\right)\\ \cdot \\ \cdot \\ \cdot \\ y\left(v,t_{m}\right) \end{array}\right]$$

The synchronous spectral intensity Φ(***v***_1_, ***v***_2_) is equal to the cross product of the dynamic spectral intensity at (*v*_1_, *v*_2_). The asynchronous spectral intensity Ψ(*v*_1_, *v*_2_) is equal to the cross product of the Hilbert-Noda matrix defined as *N*_*jk*_ for the dynamic spectral intensity at (*v*_1_, *v*_2_). Their expressions are as follows:


(2) $$\text{Φ}\left(v_{1},v_{2}\right)=\frac{1}{m-1}S{\left(v_{1}\right)}^{T}\cdot S\left(v_{2}\right)$$

(3) $$\Psi \left(v_{1},v_{2}\right)=\frac{1}{m-1}S{\left(v_{1}\right)}^{T}\cdot N\cdot S\left(v_{2}\right)$$

(4) $$\begin{array}{c} N_{jk}=\{\begin{array}{c} 0\;j=k\\ \frac{1}{\pi \left(k-j\right)}j\neq k \end{array} \end{array}$$

The product of a pair of synchronous and asynchronous correlation intensities can obtain the integrated two-dimensional correlation intensity, which is expressed asI(*v*_1_, *v*_2_) (Chen et al., 2018).


(5) $$\begin{array}{c} \text{I}\left(v_{1},v_{2}\right)=\left[\Phi \left(v_{1},v_{2}\right)\right]\cdot \left[\Psi \left(v_{1},v_{2}\right)\right]\\ =\frac{1}{{\left(m-1\right)}^{2}}\left[S{\left(v_{1}\right)}^{T}\cdot S\left(v_{2}\right)\right]\cdot \left[S{\left(v_{1}\right)}^{T}\cdot N\cdot S\left(v_{2}\right)\right] \end{array}$$

Spectral data matrix S(m × n) contains two spectra, the first is the average FT-MIR of each class, and the second is the *i*th FT-MIR spectra of each class. The synchronous 2DCOS spectra, asynchronous 2DCOS spectra and integrative 2DCOS (i2DCOS) spectra for the *i*th sample of each category can be obtained by equation (2), (3) and (4). In order to reduce the amount of calculation, save computer resources and speed up the calculation efficiency, the fingerprint area of 1,750–550 cm^–1^ was selected, and the synchronous 2DCOS, asynchronous 2DCOS and i2DCOS spectral images were automatically generated by the software Matlab2017b. The image size can be chosen according to the processing power of the computer (32 × 32 pixel, 64 × 64 pixel and 128 × 128 pixel), and the generated 2DCOS images were stored in JPEG image format with the size as 64 × 64 pixel in the corresponding folder for building ResNet model. Using the Kennard-stone algorithm, all datasets were divided into training set (60%), test set (30%), and external validation set (10%). The process of generating all types of 2DCOS spectra images is shown in Supplementary Figure 1.

### Partial Least Squares Discrimination Analysis

Partial least squares discriminant analysis is a linear supervised classification method established on the basis of the standard PLS regression algorithm. It searches for the variable with the largest covariance of the classification matrix Y from the variable matrix X. Y is divided into two categories, where Y = 1 represents that the sample belongs to a specific category, and Y = 0 represents that the sample does not belong to a specific category. Finally, the probability of each sample classified into each category is obtained. In the calculation, the observed X matrix is transformed into a set of several intermediate linear latent variables (LVs). The first n LVs are selected according to the maximum eigenvalue greater than 1. The statistical parameters of accuracy, model fitting determination coefficient R^2^, Q^2^, root mean square error of estimation (RMSEE), root mean square error of cross validation (RMSECV), and root mean square error of prediction (RMSEP) are used to evaluate the performance of the model. Permutation test was performed on the established model with a total of 50 iterations. And according to the R^2^-intercept and Q^2^-intercept results, the fitting degree of the model was verified. The process of establishing PLS-DA model was carried out on SIMCA-P+14.1 software.

### Support Vector Machine

Support vector machine is a supervised pattern recognition method that can identify unknown samples and has the ability to analyze the data with high collinearity and high noise. The libsvm-3.20 toolbox developed by the Institute of Industrial Engineering, National Taiwan University, Lin Zhiren, etc., was used to establish SVM discriminant models to identify the region and part of *P. polyphylla* var. *yunnanensis*. The 1,789 data points of the original FT-MIR spectra were used as the X variable, and the classification labels were used as the *Y* variable. The training set was used to establish discriminant models, and the text set was used to externally verify the accuracy of models. The best kernel functions *c* and *g* were obtained by cross validation of grid search method. The SVM models were implemented using Matlab software.

### Residual Neural Network

In this study, a 12-layer ResNet was established with a weight attenuation coefficient λ of 0.0001 and a learning rate of 0.01. Supplementary Table 3 showed the ResNet network parameter configuration. The model was completed by the anaconda data processing hardware platform, and MXNet was selected as the deep learning framework. The model contains two kinds of residual block, namely the identity residual block (Supplementary Figure 2) and the convolutional residual block (Supplementary Figure 3). The block is selected according to whether the dimensions of the input and output are consistent. When the dimensions of the input and output are the same, the identity residual block is used to build the model. When the input and output dimensions are inconsistent, we introduce the convolutional residual block with a convolution kernel size of 1 × 1 to match the dimensions of the input and output. The model structure is shown in Supplementary Figure 4, where the input data is synchronous 2DCOS, asynchronous 2DCOS and i2DCOS spectral images. The identification flow chart of ResNet is shown in Supplementary Figure 5. The training set is used to train the model. The Stochastic Gradient Descent (SGD) method is used to find the optimal parameters for minimizing the loss function value to obtain the optimal model. The test set is used to verify whether the performance of the final model is optimal. The external validation set is used to verify the generalization ability of the model.

## Results and Discussion

### FT-MIR Spectra Analysis

**FIGURE 3:**
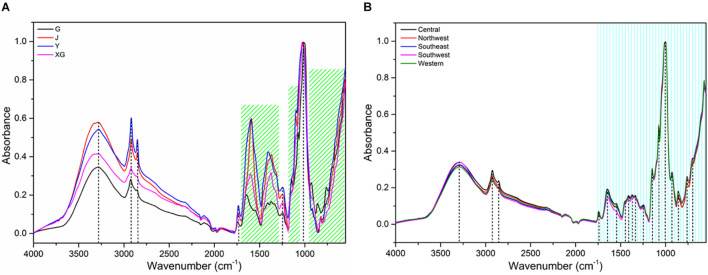
Averaged raw spectra of *Paris polyphylla* var. *yunnanensis.* **(A)** parts; **(B)** regions. The G, J, Y, and XG represent the rhizome (G), stem (J), leaf (Y) and fibrous root (XG), respectively.

Figure 3 shows the average FT-MIR spectra of four parts and five regions of PPY. 3,350, 2,940, 1,645, 1,387, 1,069, 931, 581 cm^–1^ are the main characteristic absorption peaks of PPY samples. The absorption peak of O-H stretching vibration is mainly near 3,350 cm^–1^ (Pei et al., 2018). The absorbance intensity around 2,940 cm^–1^ is related to the stretching vibration of C-H absorption of lipids (Pei et al., 2019). The absorption peak at 1,645 cm^–1^ is assigned to the C = C and C = O stretching vibration of steroid saponin and flavonoid (Wu et al., 2019). The absorption peak near 1,387 cm^–1^ is -CH_3_ symmetrical bending vibration (Yang et al., 2019). In the region of 1,300–550 cm^–1^, the absorption peaks correspond to the stretching vibration peak of C-O and the bending vibration of O-H, which belong to substances such as sugars and saponins (Wu et al., 2018). It is concluded that the main components in the plant of PPY are flavonoids, starch and glycosides.

As shown in Figure 3A, the absorption peak intensity of rhizome, stem, leaf and fibrous root is significantly different, especially the absorption peak in the band of 4,000–1,200 cm^–1^. On the whole, the order of absorption intensity of four parts is Y > J > XG > G. It may imply that the distribution and content of active components in different parts of PPY are significantly different, and the components content of non-medicinal parts (Y, J, and XG) may be higher than the medicinal parts (G), which is nearly consistent with the research results of Feng et al. (2015). However, the differences of peak shape and absorption intensity in different regions (Figure 3B) are much lower than those in different parts, which indicates that the differences within individuals may be greater than the differences between individuals, and it’s easier to identify parts than regions. Nonetheless, further modeling analysis and more studies are needed to support this conclusion.

### The Two-Dimensional Correlation Spectroscopy Spectra Images

**FIGURE 4:**
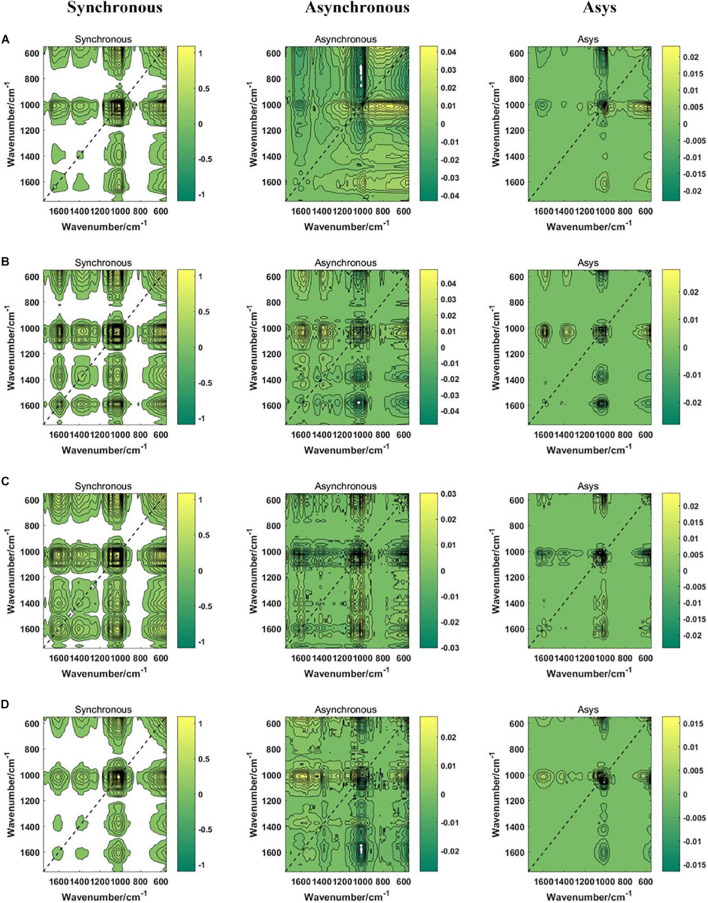
The synchronous, asynchronous and integrated 2DCOS images of parts. **(A)** rhizome; **(B)** stem; **(C)** leaf; **(D)** fibrous root. Asys images are i2DCOS images.

**FIGURE 5:**
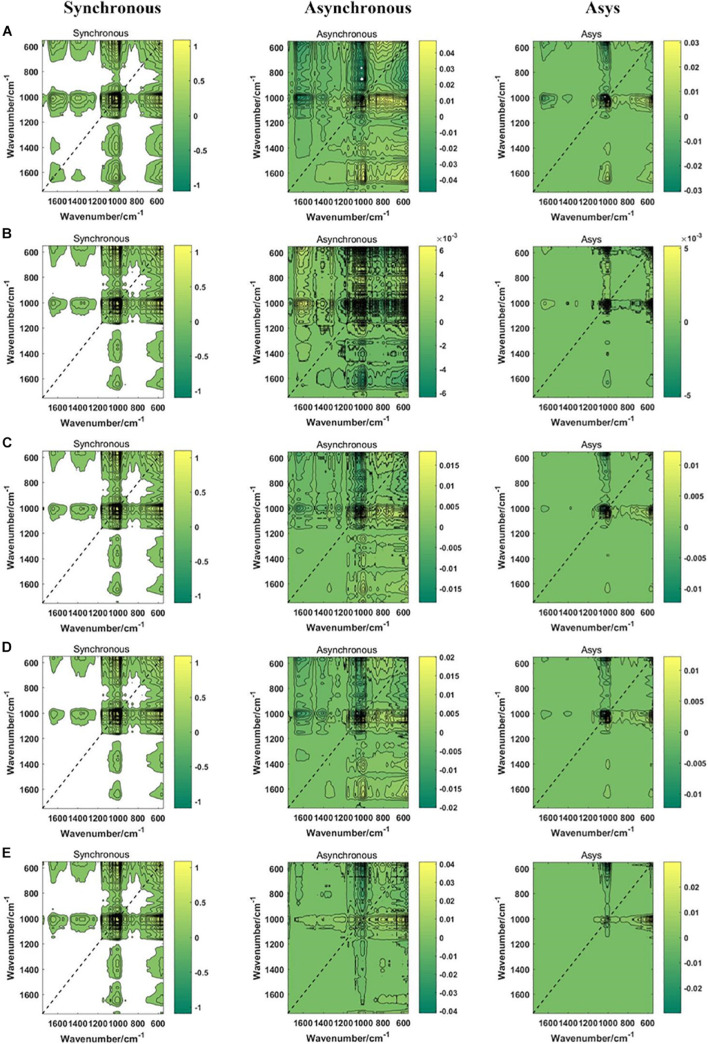
The synchronous, asynchronous and integrated 2DCOS images of regions. **(A)** central; **(B)** northwest; **(C)** southeast; **(D)** southwest; **(E)** western. Asys images are i2DCOS images.

In this study, a total of 6,135 2DCOS images were drawn, including synchronous 2DCOS, asynchronous 2DCOS and i2DCOS images of PPY in different parts (Figure 4) and different regions (Figure 5). The synchronous 2DCOS images are symmetric along diagonals, and the correlation peaks may appear on or off the diagonal. The correlation peak on the diagonal line is called the auto peak, which is expressed as the value of the auto-correlation function of spectral intensity change (Huang et al., 2003). The peaks on both sides of the diagonal are called cross peaks and represent synchronous changes of spectral signals at different wavenumbers. The asynchronous 2DCOS images characterize the asynchronous characteristics of the absorption intensity measured at two different wavenumbers. It is anti-symmetric on both sides of the diagonal, and it has only cross peaks and no automatic peaks (Noda, 1990). The i2DCOS is defined as the product of the synchronous and asynchronous two-dimensional correlation intensities. It can provide correlation spectra with equal resolution, and its characteristics are clearer than asynchronous 2DCOS (van der Maaten and Hinton, 2008). By comparing the synchronous, asynchronous and integrated 2DCOS, it is not difficult to see that the colors and lines of the synchronous images are clearer and richer, and it is easy to analyze the differences and intensity changes of auto peaks and cross peaks between different samples. However, asynchronous and integrated images are complex and changeable, and cannot be distinguished by naked eyes. This may be caused by the complex characteristics of traditional Chinese medicine. In addition, the 2DCOS images of different parts has more significant differences than that of different regions, which is consistent with the results presented by the one-dimensional spectral analysis.

In summary, synchronous 2DCOS has better performance of visual recognition. Different parts are easier to distinguish than different regions. Although 2DCOS overcame the shortcomings of one-dimensional spectral peak overlap and improved its apparent resolution, it was very difficult to recognize different parts and regions by visual analysis alone, so we need to rely on machine learning methods.

### Exploratory Analysis of t-Distributed Stochastic Neighbor Embedding

As a relatively novel non-parametric dimensionality reduction technology, t-SNE can visualize high-dimensional data to obtain the position of each data point on a two-dimensional or three-dimensional map. Its focus is to maintain the basic structure of the data matrix to reveal outliers or similarities and differences between groups of observed variables. As shown in Supplementary Figure 6, t-SNE was used in this study to conduct a preliminary visual evaluation of the spectral data sets. The ellipses in the figure represented the detailed trends of different types of samples. Supplementary Figure 6A showed the distribution of FT-MIR data sets of different parts, in which there were obvious outliers in both fibrous roots and roots. But in general, most samples could be clustered according to different category, and a few samples were mixed together. Supplementary Figure 6B showed the distribution of FT-MIR data sets of different regions, which formed a sharp contrast with the data set of different regions. The samples from the five regions were almost completely blended together. The two-dimensional visual results showed that the FT-MIR information of PPY samples in different regions was relatively similar, and it is not easy to distinguish. The results of these exploratory data analysis were consistent with the results of spectrum analysis, that is, the difference between different parts of PPY was higher than that of different regions. Obviously, in the process of data visualization, the vast majority of samples cannot be classified according to their pre-identified labels of different sources. Therefore, further in-depth modeling analysis should be considered.

### Discrimination Results of Partial Least Squares-Discriminant Analysis Model

The PLS-DA models for the parts and regions of PPY based on different sample size data sets were, respectively, established. Table 1 lists all the model parameters and the results of discrimination accuracy. From the table, we can clearly know that the models of different parts, different regions and different sample sizes have significant differences in the identification ability and model performance. In addition, in order to assess whether the PLS-DA model has an over-fitting problem, a permutation test was performed on all models. Generally, if the intercept of R^2^ is less than 0.4, there is no risk of over-fitting. Supplementary Figure 7 shows the results of the permutation test of five classification models (PLS-DA model cannot be established based on the low sample size data of the region). The results show that the R^2^ intercepts of the five models are all less than 0.4, and there is no risk of over-fitting. The confusion matrices of the established PLS-DA models based on the data set of parts and regions are shown in Supplementary Tables 4, 5, respectively.

**TABLE 1:** Parameters for PLS-DA models in parts and regions discrimination based on three levels of data sets.

Data	Model	LVs	R^2^	Q^2^	RMSEE	RMSECV	RMSEP	Accuracy (%)	
								Training set	Test set
**Parts**	PLS-DA-L	1	0.198	0.159	0.374135	0.37687	0.295164	51.52	55.56
	PLS-DA-M	11	0.899	0.831	0.143237	0.167712	0.0758287	99.39	100
	PLS-DA-H	11	0.918	0.887	0.120499	0.138129	0.0669199	99.39	100
**Regions**	PLS-DA-L	/	/	/	/	/	/	/	/
	PLS-DA-M	14	0.584	0.333	0.349237	0.441024	0.325103	87.92	88.46
	PLS-DA-H	20	0.698	0.544	0.266242	0.351347	0.266231	95.34	92.22

First of all, from the models based on different parts of the data set, we can see that the R^2^ and Q^2^ of the PLS-DA-L model are only 0.198 and 0.159, respectively, which are both lower than 0.5, and the recognition accuracy of the test set is only 55.56%. Therefore, the model based on the low sample size data set has poor performance and low discrimination ability, and cannot realize the discrimination of different parts of PPY. The PLS-DA-M and PLS-DA-H models based on the data sets of parts have high R^2^ and Q^2^ values greater than 0.8 and low RMSEE, RMSECV and RMSEP values. The accuracy of the test sets of the two models is 100%, which has a very good recognition performance.

Secondly, as shown in the table, the PLS-DA-L model based on regions data cannot be fitted. This result may be related to the amount of data being too small or the data is not preprocessed. Although the PLS-DA-M model has a test set accuracy rate of 88.46%, the model performance is poor with low Q^2^ and high RMSEE, RMSECV, and RMSEP values. The PLS-DA-H model is better than the low sample size model and the medium sample size model in terms of model performance and recognition accuracy, so that it can well identify PPY in different regions.

Finally, from the perspective of sample size, whether it is PLS-DA models based on part data or models based on region data, the recognition performance is dependent on the sample size. And it shows that the larger the sample size, the better the model performance and the stronger the recognition ability. However, with the increase of the sample size, the recognition efficiency of the models will be greatly reduced. In addition, through comparison, it can be concluded that the PLS-DA models based on part data is better than that based on region data, regardless of model parameter results or recognition accuracy.

### Discrimination Results of Support Vector Machine Model

Support vector machine is a supervised classification tool. It searches for the optimal separation hyperplane between different data categories by maximizing the distance between the classification hyperplane and various sample points. SVM contains two parameters, *c* is used as a penalty parameter, which can control the generalization ability of the model and reduce the over-fitting phenomenon, and the kernel function parameter *g* is related to the stability of the model. Supplementary Figures 8, 9 are the optimal separation hyperplane graph and classification result graph of the SVM model based on parts and regions data, respectively. The detailed results of the six SVM models are shown in Table 2. Best *c* and Best *g*, respectively, represent the best penalty parameter and kernel function parameter of the model.

**TABLE 2:** The accuracy of SVM models for parts and regions identification based on three levels of data sets.

Data	Model	Best *c*	Best *g*	Accuracy (%)	
				Training set	Test set
**Parts**	SVM-L	2,048.00	0.000043	72.73	100.00
	SVM-M	181.02	0.00069	98.18	100.00
	SVM-H	5.66	0.016	99.39	100.00
**Regions**	SVM-L	1.00	0.10	0.00	46.15
	SVM-M	11,585.24	0.00017	87.92	92.31
	SVM-H	46,340.95	0.000031	94.17	97.28

The accuracy difference between the training set and the test set of the SVM-L model based on part data and region data is more than 20%, while the accuracy of the training set and the test set of the SVM-M and SVM-H models based on part data and region data is less than 5%. This shows that the reliability of the SVM models established with low sample size data is poor. The SVM-M and SVM-H models based on part data both have high identification accuracy and low Best *c* value, so the model performance is good and have the ability to identify different parts of PPY. However, although the SVM-M and SVM-H models based on region data have high identification accuracy, their Best *c* values are abnormally high, indicating that the performance of the two models is poor and there may be over-fitting, which can’t well identify the PPY in different regions. The above results show that although a larger sample size can improve the identification accuracy of the SVM model, the establishment of a high-performance model cannot be achieved for data that has not been preprocessed and has small differences between different categories. In addition, as with the results of the PLS-DA model, it is easier to identify the parts of PPY than the regions.

In conclusion, although the SVM model has the advantage of solving the problems of small sample, nonlinear and high-dimensional data (Noble, 2006), the unpreprocessed small sample data in this study is not applicable to the SVM model, indicating that data preprocessing is very necessary to improve the discrimination performance of traditional models such as SVM. In addition, a larger sample size increases the over-fitting risk of SVM model while improving the recognition accuracy, which leads to poor model performance and low reliability.

### Discrimination Results of Residual Neural Network Model

**FIGURE 6:**
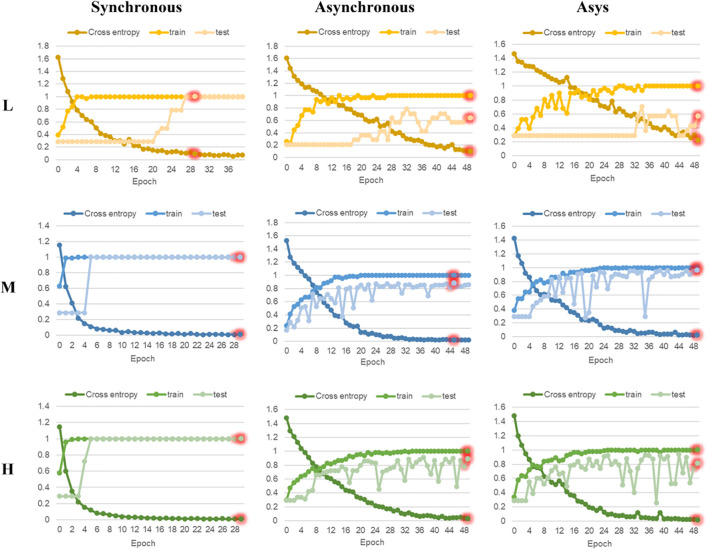
The accuracy curves and cross-entropy cost function of ResNet models based on part data with different sample size. L, low sample size; M, medium sample size; H, high sample size.

**FIGURE 7:**
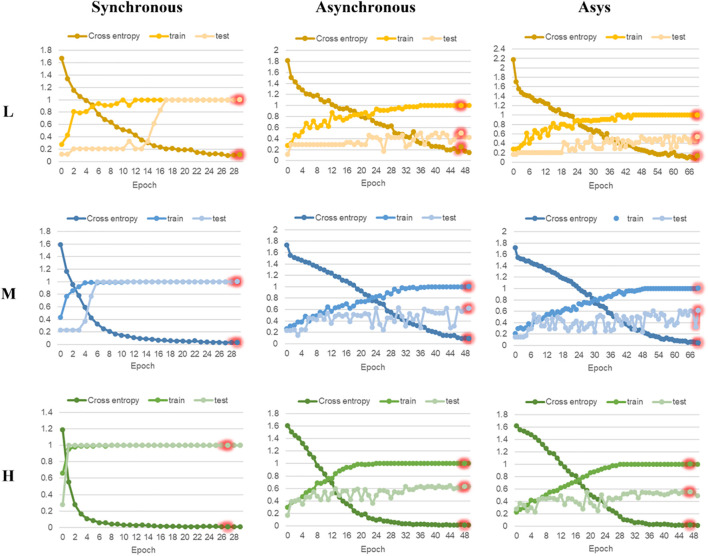
The accuracy curves and cross-entropy cost function of ResNet models based on region data with different sample size. L, low sample size; M, medium sample size; H, high sample size.

In this research, ResNet models based on 2DCOS images (including synchronous, asynchronous and integrated images) of FT-MIR were established. Figures 6, 7 are the results of 18 ResNet models based on the data sets of parts and regions, respectively, showing the accuracy curves and cross-entropy cost function curves. The accuracy curves, includes the training set and the test set, were used to evaluate the discrimination ability of the model. The closer its value is to 1, the stronger the discrimination ability of the model. The cross-entropy loss function was used to explain the convergence effect of the model. The closer its value is to zero, the better the convergence effect of the model. In addition, the external validation set was classified using the models established above, and the classification result of the external validation set of different parts and regions was shown in the confusion matrix in Supplementary Figures 10, 11, respectively. External validation is used to judge and evaluate the pros and cons of the model to ensure the stability of the established model. Table 3 summarized the result parameters of all models, including accuracy (training set, test set and external validation set), epoch, and loss value.

**TABLE 3:** The accuracy of ResNet models for parts and regions identification based on three levels of data sets.

Data	Code	Type	Epoch	Loss value	Accuracy		
					Train (%)	Test (%)	External validation (%)
**Parts**	Resnet-L	**Synchronous**	**29**	**0.091**	**100**	**100**	**100**
		Asynchronous	49	0.102	100	64	100
		Asys	49	0.219	100	57	100
	Resnet-M	**Synchronous**	**29**	**0.012**	**100**	**100**	**100**
		Asynchronous	45	0.021	100	88	87.5
		Asys	49	0.021	100	96	100
	Resnet-H	**Synchronous**	**29**	**0.009**	**100**	**100**	**100**
		Asynchronous	49	0.027	100	89	90
		Asys	49	0.017	100	81	88
**Regions**	Resnet-L	**Synchronous**	**29**	**0.114**	**100**	**100**	**100**
		Asynchronous	47	0.248	100	50	25
		Asys	69	0.132	100	54	37.5
	Resnet-M	**Synchronous**	**29**	**0.030**	**100**	**100**	**100**
		Asynchronous	49	0.088	100	62	56.4
		Asys	69	0.045	100	61	66.7
	Resnet-H	**Synchronous**	**27**	**0.009**	**100**	**100**	**100**
		Asynchronous	48	0.011	100	63	62.7
		Asys	47	0.020	100	55	64

*Note: The bold value are the optimal results of models under the certain data set.*

Comparing the models based on synchronous, asynchronous and integrated 2DCOS images, we can get that the model of synchronous 2DCOS images has the best discrimination effect, and the accuracy of the training set, test set and external verification set is 100%. The modeling results are consistent with the results of image vision analysis, that is, the synchronized 2DCOS images have clearer characteristic peaks and can better characterize different types of samples. Comparing the models with low, medium and high sample sizes showed that the ResNet model had no dependence on the sample size, and there was no obvious rule between the identification accuracy and the sample size. However, too small sample size will lead to poor performance and over-fitting of model. This result can be derived from the identification results of low sample size models based on asynchronous and integrated 2DCOS images. The difference of identification accuracy between the external validation set and the test set was large, and the loss value of models was significantly higher than that of the medium sample size and high sample size models. In addition, the accuracy curves of the training set and test set of the medium sample size and high sample size models showed a consistent upward trend, which also showed that these two types of models had no risk of over-fitting and were robust. However, the accuracy curve of the training set and the test set of the low sample size model had a poor consistency in the upward trend, even for the optimal model of synchronous 2DCOS images, which indicated that the low sample size would reduce the performance of the ResNet model. Finally, on the whole, the recognition effect of the ResNet model based on the part data set was better than that of the ResNet model based on the region data set.

In summary, the recognition accuracy of the models based on synchronous 2DCOS images is the best, which is almost not affected by sample size, part, region and other factors, and is most suitable for the identification of medicinal plants. However, too small sample size does have a small negative impact on the performance of the ResNet model. Therefore, it is worth thinking about how to use an appropriate method to solve the negative impact of low samples on model performance. This is conducive to solving the identifying problem of research subjects with a small sample size. These research objects have very limited data, and it is expensive or impossible to obtain more data, such as scarce and precious animal and plant resources.

### Comparison Analysis of Models

**FIGURE 8:**
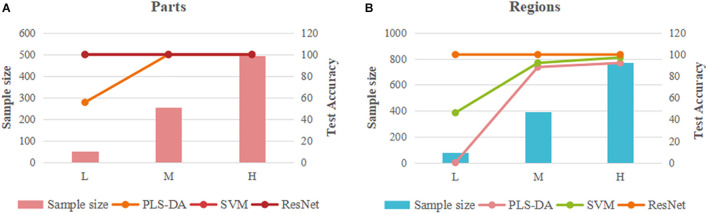
Comparison of the overall identification performance of PLS-DA, SVM and ResNet models. **(A)** parts; **(B)** regions.

Partial least squares discriminant analysis, SVM, and ResNet models showed significant differences in their ability to identify the parts and regions of the PPY, the responses to different sample sizes, and the comprehensive performance of models. As shown in Figure 8, we have made a visual comparison of three type of models.

In terms of the identification ability of parts and regions, the three types of models show consistent results, that is, the identification ability of parts is better than that of regions, which indicates that the difference of parts data of PPY is greater than that of regions data. This result implies that the difference in component within the sample may be greater than that between samples. This causes us to think about the resource evaluation and the effective development and utilization of the non-medicinal parts of PPY. In addition to the evaluation of the advantages and disadvantages of the medicinal parts between individuals in different origins, the development and utilization of non-medicinal parts within individuals is also very worthy of attention.

From the perspective of different sample sizes, the three models have different responses to low, medium, and high sample size data. The PLS-DA model has a very significant sample size dependence. As the sample size increases, the discrimination ability and the performance of the model have been significantly improved. It can be concluded that the overall performance of the PLS-DA model is positively correlated with the sample size. This result is confirmed by two types of models based on part and region data, which greatly reduces the chance. There is a certain correlation between the merits and demerits of SVM model and the sample size, but not a complete positive or negative correlation. The identification accuracy of the model increases with the increase of the sample size, while the performance of the model based on region data evaluated by parameters will deteriorate with the increase of the sample size. It can be concluded from this study that there are two important factors affecting the overall performance of SVM model, one is the quality of data itself, the other is the sample size. The ResNet model based on the synchronous 2DCOS images has a very perfect overall discrimination performance, both in terms of the discrimination accuracy and the model parameters. It is not limited by the sample size and is almost unaffected by the data itself. Whether it is based on easy-to-identify part data or region data with small differences, it can achieve 100% recognition accuracy.

In summary, the PLS-DA model has the strongest dependence on the sample size, followed by SVM, and the ResNet model based on synchronized 2DCOS images has almost no dependence on the sample size. In addition, the traditional pattern recognition model is also affected by the quality of data itself. Therefore, the ResNet model based on synchronized 2DCOS images occupies an absolute advantage in the identification of medicinal plants. The model is universal and does not require preprocessing or artificial extraction of characteristic variables. It has good discrimination accuracy regardless of the sample size or the quality of the data.

## Conclusion

In this study, we used three kinds of models to identify the part and region of PPY. PLS-DA and SVM are traditional pattern recognition models, which have been widely used in the past research. ResNet model is a representative dominant model in deep learning. The effects of different types of data and different sample sizes on the discrimination ability and performance of the three models were discussed without any data preprocessing. By comparing the ability of the traditional model and the deep learning model for the identification of PPY, we found that the identification performance of PLS-DA and SVM models was easily affected by the data type, sample size and other factors, and the overall identification ability of both models was not as good as the ResNet model based on synchronous 2DCOS images. Different from the previous single theory or single model analysis, this study verified the superiority of deep learning model in the identification research of medicinal plant resources from the actual and multiple perspectives.

## Data Availability Statement

The raw data supporting the conclusion of this article will be made available by the authors, without undue reservation.

## Author Contributions

JY: conceptualization, software, formal analysis, writing–original draft preparation, and writing—review and editing. WL: methodology, resources, and software. YW: supervision, project administration, and funding acquisition. All authors have read and agreed to the published version of the manuscript.

## Conflict of Interest

The authors declare that the research was conducted in the absence of any commercial or financial relationships that could be construed as a potential conflict of interest.

## Publisher’s Note

All claims expressed in this article are solely those of the authors and do not necessarily represent those of their affiliated organizations, or those of the publisher, the editors and the reviewers. Any product that may be evaluated in this article, or claim that may be made by its manufacturer, is not guaranteed or endorsed by the publisher.
