# Supplementary material for: Superiority Verification of Deep Learning in the Identification of Medicinal Plants: Taking Paris polyphylla var. yunnanensis as an Example
Source: Front Plant Sci. 2021 Sep 22;12:752863. doi: 10.3389/fpls.2021.752863 (PMC8493076; doi:10.3389/fpls.2021.752863)
Supplement: Supplementary file 1 [file Data_Sheet_1.docx]

**[Supplementary](D:/%E4%B8%8B%E8%BD%BD/%E7%BD%91%E6%98%93%E6%9C%89%E9%81%93%E8%AF%8D%E5%85%B8/Dict/8.9.6.0/resultui/html/index.html#/javascript:;) [materials](D:/%E4%B8%8B%E8%BD%BD/%E7%BD%91%E6%98%93%E6%9C%89%E9%81%93%E8%AF%8D%E5%85%B8/Dict/8.9.6.0/resultui/html/index.html#/javascript:;)**

Figure S1 The generation processes of all types of 2DCOS images

Figure S2 The schematic diagram of identity block

Figure S3 The schematic diagram of convolutional block

Figure S4 The structure of ResNet model used in this research

Figure S5 The discrimination strategy of *Paris polyphylla* var. *yunnanensis* based on ResNet

Figure S6 The t-SNE visualization distribution of *Paris polyphylla* var. *yunnanensis.* (A), parts;

Figure S7 Permutation test results of the partial least square discrimination analysis model based on part and region data with different sample size

Figure S8 Optimal separation of hyperplane (left) and classification result (right) of SVM model based on part data with different sample size. (A), low sample size; (B) medium sample size; (C) high sample size

Figure S9 Optimal separation of hyperplane (left) and classification result (right) of SVM model based on region data with different sample size. (A), low sample size; (B) medium sample size; (C) high sample size

Figure S10 The confusion matrix of ResNet models based on part data with different sample size. (L), low sample size; (M), medium sample size; (H), high sample size

Figure S11 The confusion matrix of ResNet models based on region data with different sample size. (L), low sample size; (M), medium sample size; (H), high sample size

Table

Table S1 The information of *Paris polyphylla* var. *yunnanensis* samples

Table S2 The distribution of sample sizes at three levels from different parts and regions data

Table S3 ResNet network parameter configuration

Table S4 Confusion matrix of PLS-DA models based on the three levels of parts data

Table S5 Confusion matrix of different PLS-DA models based on the three levels of regions data


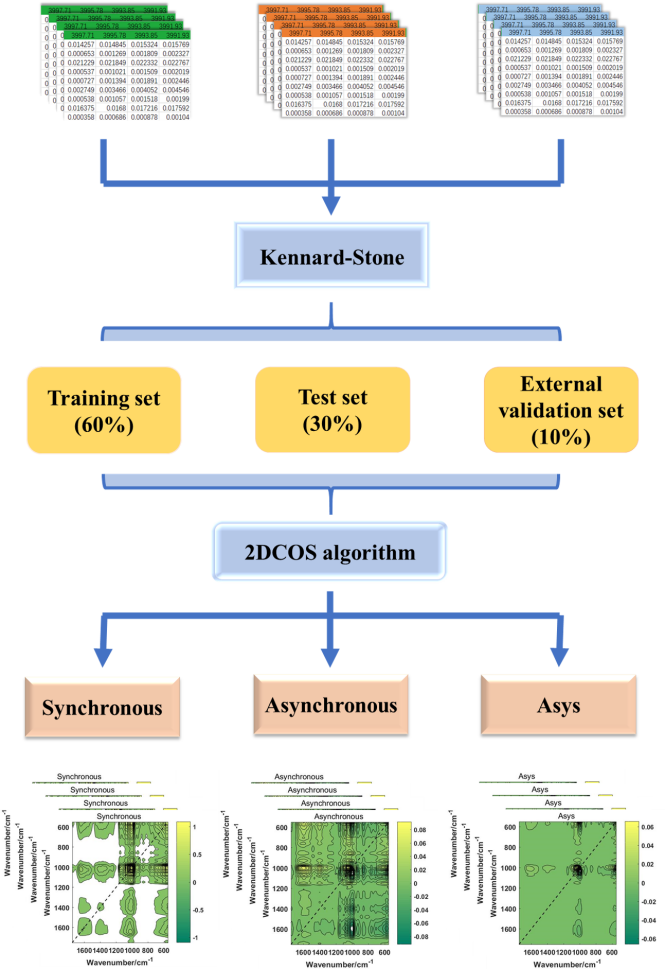


Figure S1 The generation processes of all types of 2DCOS images


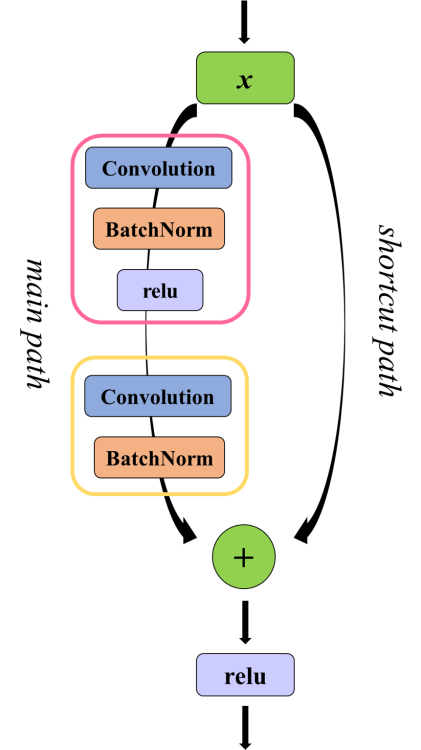


Figure S2 The schematic diagram of identity block


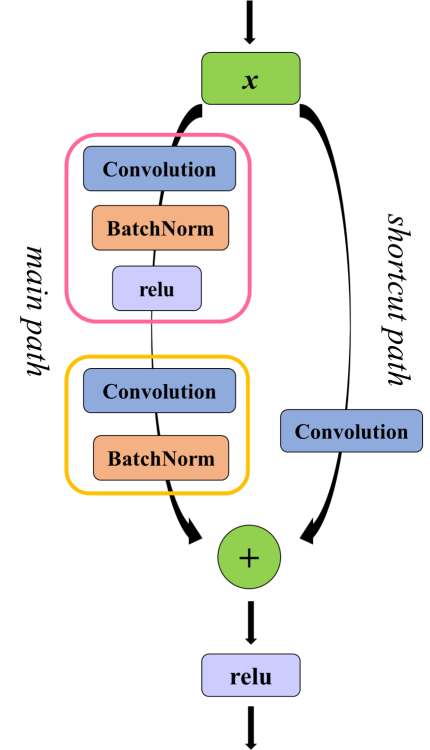


Figure S3 The schematic diagram of convolutional block


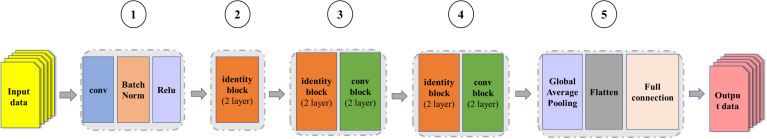


Figure S4 The structure of ResNet model used in this research


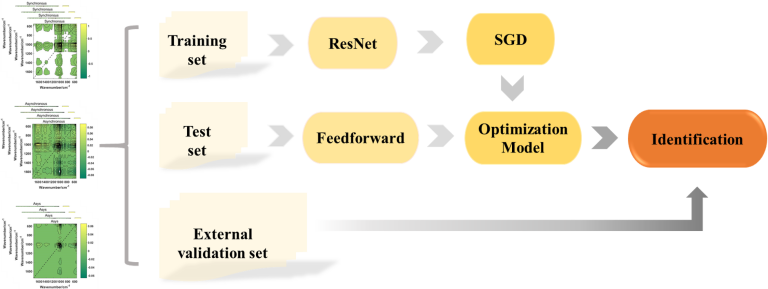


Figure S5 The discrimination strategy of *Paris polyphylla* var. *yunnanensis* based on ResNet


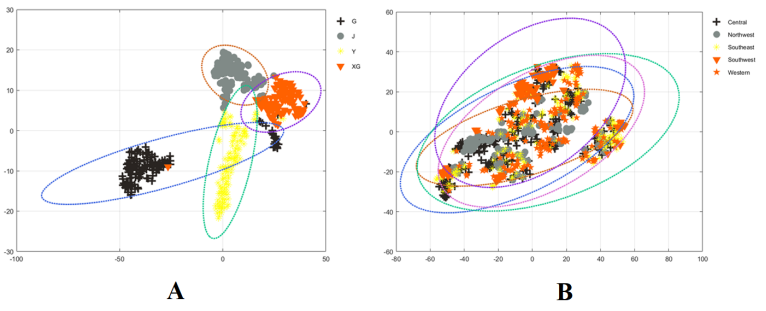


Figure S6 The t-SNE visualization distribution of *Paris polyphylla* var. *yunnanensis.* (A), parts; (B), regions


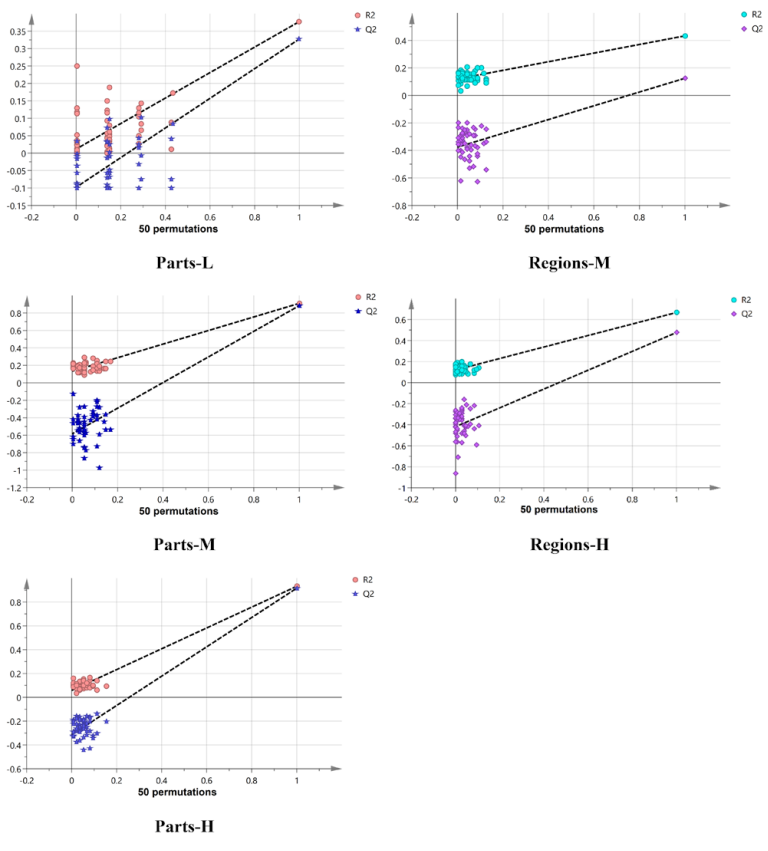


Figure S7 Permutation test results of the partial least square discrimination analysis model based on part and region data with different sample size


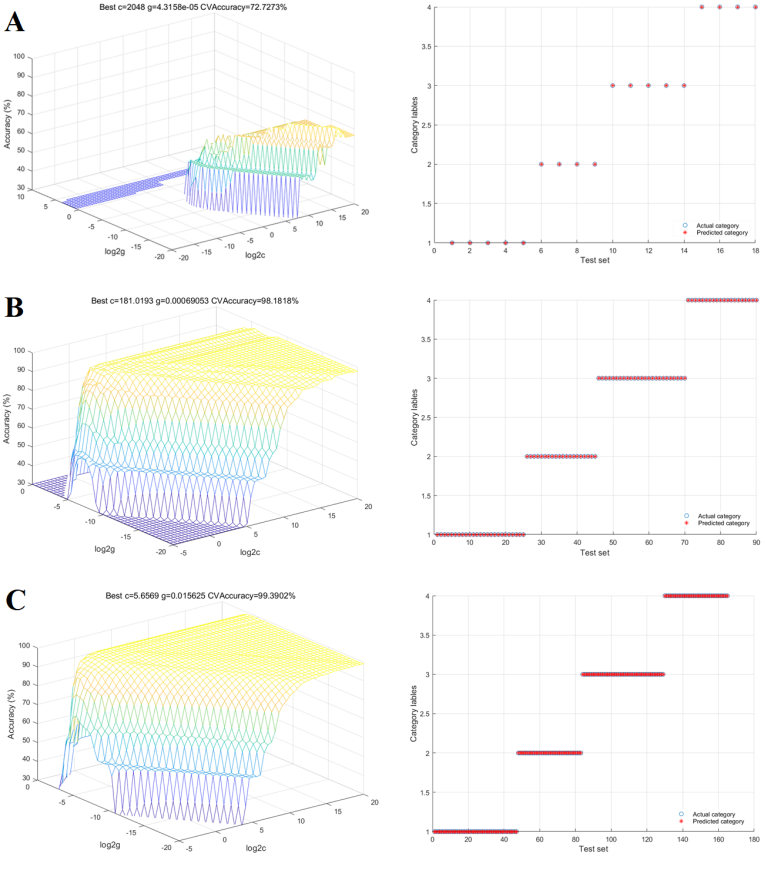


Figure S8 Optimal separation of hyperplane (left) and classification result (right) of SVM model based on part data with different sample size. (A), low sample size; (B) medium sample size; (C) high sample size


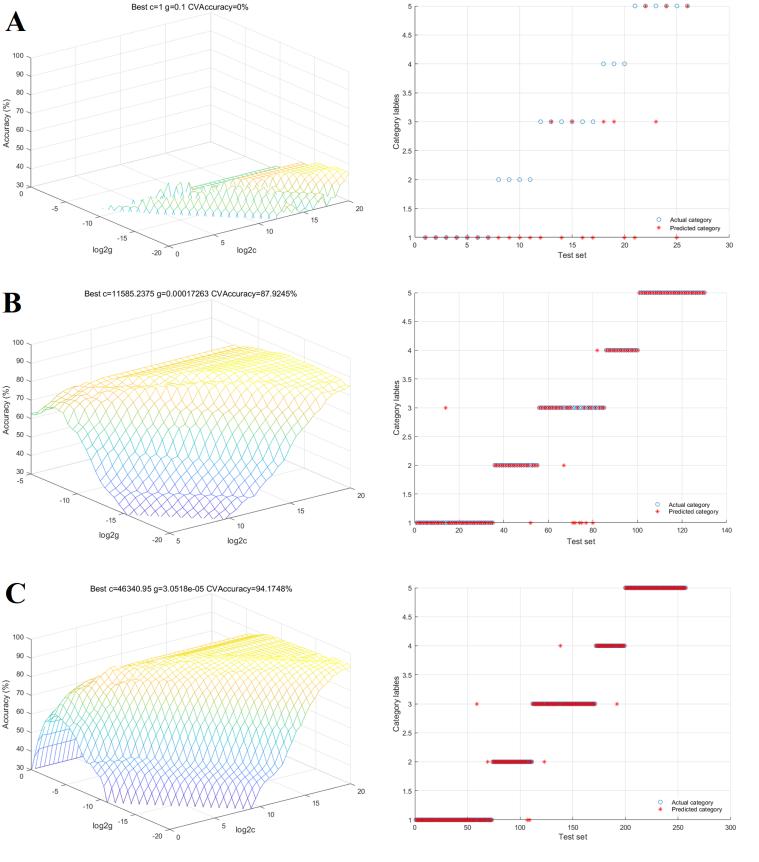


Figure S9 Optimal separation of hyperplane (left) and classification result (right) of SVM model based on region data with different sample size. (A), low sample size; (B) medium sample size; (C) high sample size


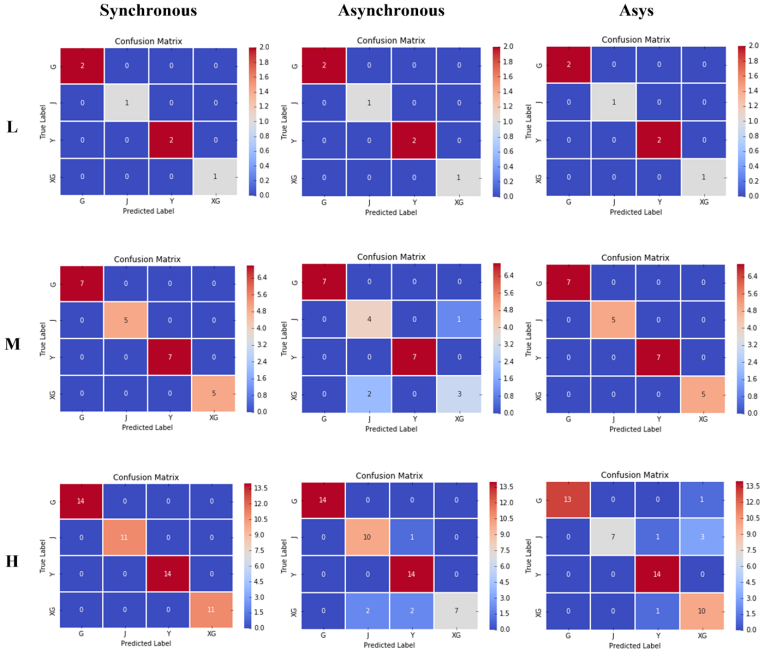


Figure S10 The confusion matrix of ResNet models based on part data with different sample size. (L), low sample size; (M), medium sample size; (H), high sample size


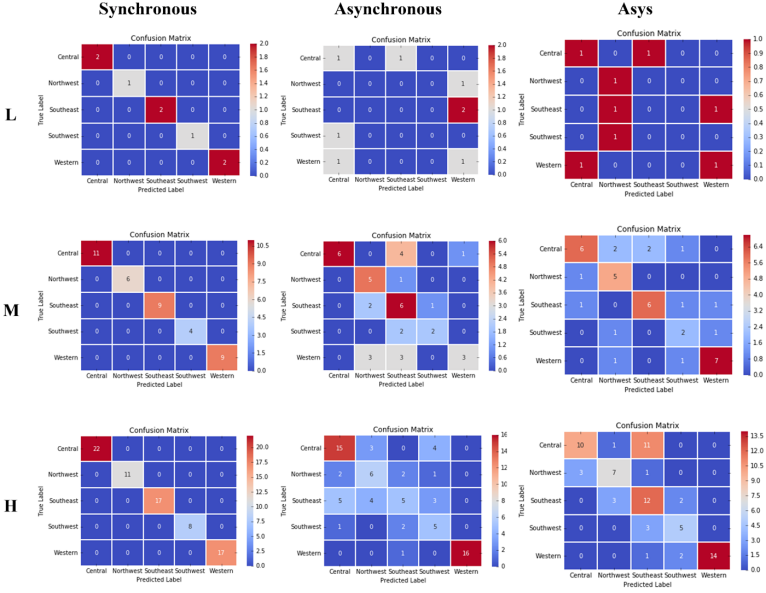


Figure S11 The confusion matrix of ResNet models based on region data with different sample size. (L), low sample size; (M), medium sample size; (H), high sample size

Table S1 The information of *Paris polyphylla* var. *yunnanensis* samples

| Regions | Sites | Longitude | Latitude | Elevation | Sample size ( Parts ) | | | |
| --- | --- | --- | --- | --- | --- | --- | --- | --- |
|  |  |  |  |  | Rhizome | Stem | Leaf | Fibrous root |
| Western | Baoshan | E98°30′42″ | N24°36′4″ | 2092.80 | 65 | / | / | / |
|  | Dali | E100°20′30″ | N25°18′36″ | 2174.36 | 80 | / | / | / |
|  | Dehong | E98°32′32″ | N24°26′01″ | 1621.14 | 31 | 14 | 29 | 14 |
| Central | Kunming | E102°44′21″ | N25°11′30″ | 2224.69 | 46 | / | / | / |
|  | Yuxi | E102°26′44″ | N24°25′58″ | 2100.00 | 111 | 93 | 108 | 93 |
|  | Chuxiong | E101°19′02″ | N25°31′30″ | 2148.84 | 63 | / | / | / |
| Northwest | Nujiang | E99°20′55″ | N26°32′02″ | 2580.41 | 21 | / | / | / |
|  | Lijiang | E100°11′22″ | N26°52′39″ | 2403.27 | 92 | / | / | / |
| Southwest | Lincang | E100°14′22″ | N24°34′11″ | 1186.81 | 40 | / | / | / |
|  | Puer | E100°23′47″ | N23°25′01″ | 1072.21 | 45 | / | / | / |
| Southeast | Honghe | E103°1′20″ | N23°0′27″ | 1628.68 | 90 | / | / | / |
|  | Wenshan | E104°4′07″ | N23°14′33″ | 1766.11 | 88 | / | / | / |

Table S2 The distribution of sample sizes at three levels from different parts and regions data

| Type | Total | L (10%) | M (50%) | H (100%) |
| --- | --- | --- | --- | --- |
| Parts | 493 | 51 | 255 | 493 |
| Regions | 772 | 79 | 395 | 772 |

Note: Low sample size, L; Medium sample size, M; High sample size, H

Table S3 ResNet network parameter configuration

| Layer name | Kernel size | Channels | Strides | Block numbers | Block name |
| --- | --- | --- | --- | --- | --- |
| Conv1 | 3×3 | 32 | 1 | ×1 | Identity Block |
|  |  |  |  |  |  |
| Conv2_x | 3×3 | 32 | 1 | ×1 | Identity Block |
|  | 3×3 | 32 | 1 |  |  |
|  |  |  |  |  |  |
| Conv3_x | 3×3 | 32 | 1 | ×1 | Identity Block |
|  | 3×3 | 32 | 1 |  |  |
|  |  |  |  |  |  |
|  | 3×3 | 64 | 2 | ×1 | Conv Block |
|  | 3×3 | 64 | 1 |  |  |
|  | 1×1 | 64 | 2 |  |  |
|  |  |  |  |  |  |
| Conv4_x | 3×3 | 64 | 1 | ×1 | Identity Block |
|  | 3×3 | 64 | 1 |  |  |
|  |  |  |  |  |  |
|  | 3×3 | 128 | 2 | ×1 | Conv Block |
|  | 3×3 | 128 | 1 |  |  |
|  | 1×1 | 128 | 2 |  |  |
|  |  |  |  |  |  |
| Output | Global average pooling, Flatten, Full Connection, Softmax | | | | |

Table S4 Confusion matrix of PLS-DA models based on the three levels of parts data

| Models | Class | Training set | | | |  | Test set | | | |
| --- | --- | --- | --- | --- | --- | --- | --- | --- | --- | --- |
|  |  | G | J | Y | XG |  | G | J | Y | XG |
| PLS-DA-L | G | **9** | 0 | 1 | 0 |  | **5** | 0 | 0 | 0 |
|  | J | 1 | **0** | 6 | 0 |  | 0 | **0** | 4 | 0 |
|  | Y | 1 | 0 | **8** | 0 |  | 0 | 0 | **5** | 0 |
|  | XG | 5 | 0 | 2 | **0** |  | 4 | 0 | 0 | **0** |
|  |  |  |  |  |  |  |  |  |  |  |
| PLS-DA-M | G | **50** | 0 | 0 | 0 |  | **25** | 0 | 0 | 0 |
|  | J | 0 | **35** | 0 | 0 |  | 0 | **20** | 0 | 0 |
|  | Y | 0 | 0 | **45** | 0 |  | 0 | 0 | **25** | 0 |
|  | XG | 1 | 0 | 0 | **34** |  | 0 | 0 | 0 | **20** |
|  |  |  |  |  |  |  |  |  |  |  |
| PLS-DA-H | G | **94** | 0 | 0 | 1 |  | **47** | 0 | 0 | 0 |
|  | J | 0 | **71** | 0 | 0 |  | 0 | **36** | 0 | 0 |
|  | Y | 0 | 0 | **91** | 0 |  | 0 | 0 | **46** | 0 |
|  | XG | 1 | 0 | 0 | **70** |  | 0 | 0 | 0 | **36** |

Table S5 Confusion matrix of different PLS-DA models based on the three levels of regions data

| Models | Class | Training set | | | | |  | Test set | | | | |
| --- | --- | --- | --- | --- | --- | --- | --- | --- | --- | --- | --- | --- |
|  |  | Central | Northwest | Southeast | Southwest | Western |  | Central | Northwest | Southeast | Southwest | Western |
| PLS-DA-L | Central | **15** | 0 | 0 | 0 | 0 |  | **7** | 0 | 0 | 0 | 0 |
|  | Northwest | 8 | **0** | 0 | 0 | 0 |  | 4 | **0** | 0 | 0 | 0 |
|  | Southeast | 12 | 0 | **0** | 0 | 0 |  | 6 | 0 | **0** | 0 | 0 |
|  | Southwest | 6 | 0 | 0 | **0** | 0 |  | 3 | 0 | 0 | **0** | 0 |
|  | Western | 12 | 0 | 0 | 0 | **0** |  | 6 | 0 | 0 | 0 | **0** |
|  |  |  |  |  |  |  |  |  |  |  |  |  |
| PLS-DA-M | Central | **57** | 7 | 9 | 2 | 0 |  | **26** | 0 | 6 | 3 | 0 |
|  | Northwest | 3 | **36** | 0 | 1 | 0 |  | 0 | **20** | 0 | 0 | 0 |
|  | Southeast | 6 | 0 | **54** | 0 | 0 |  | 3 | 2 | **24** | 1 | 0 |
|  | Southwest | 1 | 0 | 2 | **26** | 1 |  | 0 | 0 | 0 | **15** | 0 |
|  | Western | 0 | 0 | 0 | 0 | **60** |  | 0 | 0 | 0 | 0 | **30** |
|  |  |  |  |  |  |  |  |  |  |  |  |  |
| PLS-DA-H | Central | **138** | 0 | 6 | 3 | 0 |  | **66** | 2 | 4 | 1 | 0 |
|  | Northwest | 3 | **71** | 1 | 0 | 0 |  | 2 | **36** | 0 | 0 | 0 |
|  | Southeast | 3 | 1 | **113** | 2 | 0 |  | 4 | 0 | **54** | 2 | 0 |
|  | Southwest | 2 | 0 | 2 | **52** | 1 |  | 0 | 0 | 5 | **23** | 0 |
|  | Western | 0 | 0 | 0 | 0 | **117** |  | 0 | 0 | 0 | 0 | **58** |
